# Group C/G Streptococcal Pharyngitis and Acute Rheumatic Fever in Auckland, New Zealand, 2010–2016

**DOI:** 10.1111/jpc.70440

**Published:** 2026-05-18

**Authors:** Jane Oliver, Susan Jack, Julie Bennett, Michael G. Baker

**Affiliations:** ^1^ The Kids Research Institute Australia Nedlands Western Australia Australia; ^2^ National Public Health Service Dunedin New Zealand; ^3^ University of Otago Wellington Wellington New Zealand

In our 2021 article, we reported that *Streptococcus pyogenes* (Strep A) detection, both in throat swabs and skin swabs, was associated with an increased risk of acute rheumatic fever (ARF) [1]. This elevated risk was concentrated during the 8–90 days following swab collection. While Strep A pharyngitis is a well‐established driver of ARF, we provided the first population‐level evidence that Strep A skin infections can independently trigger ARF [1, 2].

There is occasional evidence in the international literature that infection with other Lancefield groups, notably Group C and G, may also trigger ARF, although this exposure does not appear to be a major driver of disease [3]. It is, however, important to investigate this possibility to help assess the clinical significance of superficial Group C and G infections.

In Aotearoa New Zealand, ARF almost exclusively affects Indigenous Māori and Pacific children. The highest disease burden is in South Auckland, where Māori and Pacific people make up a relatively high proportion of the population [4]. Our analysis included 1 866 981 throat and skin swabs collected in the Auckland region during 2010–2017. Swabs were cultured at Awanui Labs (formerly Labtests), the accredited community pathology laboratory service provider for the region. All ARF diagnoses corresponding to hospitalisations from 1988 to 2018 inclusive were identified in the national minimum dataset (NMDS; ICD‐10: I00‐I02 and ICD‐9: 390–392), as were all rheumatic heart disease diagnoses (ICD‐10: I05‐I09 and ICD‐9: 393–398). Linking swab culture results with NMDS data revealed that Māori and Pacific people had a five‐fold increased risk of being hospitalised with ARF for the first time during the 8–90 day period following collection of a Strep A positive throat swab or skin swab. Reviewing this time period maximised the sensitivity for detecting potentially causative infections [1].

From 2010 to 2016 inclusive, of the 566 swabs collected during the year preceding an initial ARF hospitalisation, no skin swabs, and only 33 throat swabs (5.8%), produced group C or group G streptococci (i.e., Strep C/G positive; including one where Strep A was also cultured). The risk of ARF was increased during the year following Strep C/G positive throat swab collection (RR 1.41, RR 0.99‐2.01), and particularly during the 6–12 month period following collection. Importantly, the risk was not markedly elevated during the critical 8–90 day period, as observed with Strep A pharyngitis and skin infections. Furthermore, this association was not observed in demographic groups with the highest rates of ARF (i.e., Māori and Pacific children aged 5–14 years). Confidence intervals were wide given low Strep C/G positive swab numbers (Table 1).

**TABLE 1:** Risk of ARF following a Group C/G positive throat swab compared with a Strep A & Group C/G negative swab, Auckland, Aotearoa New Zealand, 2010–2016.

	ARF rate following Strep C/G culture‐positive swab			ARF rate following Strep C/G culture‐negative swab			Rate ratio of ARF in Strep C/G culture positive to ARF in Strep A & Strep C/G negative swabs	
	ARF cases following Strep C/G +ve swab	Total Strep C/G +ve swabs	ARF rate per 100 000 Strep C/G +ve swabs^1^	ARF cases following Strep A & Strep C/G −ve swab	Total Strep A & Strep C/G −ve swabs	ARF rate per 100 000 Strep A & Strep C/G −ve swabs^1^	RR	95% Confidence Interval
Risk of developing ARF for total Auckland population during specified time windows								
ARF 1–365 days of swab	33	63 844	51.7	378	1 029 680	36.7	1.41	0.99–2.01
ARF 181–365 days of swab	18	63 844	56.4	176	1 029 680	34.2	**1.65**	**1.02**–**2.67** *
ARF 91–180 days of swab	7	63 844	43.9	100	1 029 680	38.8	1.13	0.52–2.43
ARF 1–90 days of swab	8	63 844	50.1	102	1 029 680	39.6	1.27	0.62–2.59
ARF 1–7 days of swab	2	63 844	162.9	30	1 029 680	151.5	1.08	0.26–4.50
ARF 8–90 days of swab	6	63 844	41.9	74	1 029 680	32.1	1.31	0.57–3.01

* Bold typeface indicates result is statistically significant.

A further reason why Strep C/G infection is unlikely to be an important initiator of ARF is the low prevalence of these infections. From 2010 to 2016 inclusive, only 5.1% of the 1 257 058 throat swabs cultured at Awanui Labs were Strep C/G positive [5], as were 1.5% of the 377 410 skin swabs [2].

The elevated risk of ARF within 6–12 months of Strep C/G throat swab detection may simply indicate that people who experience these infections are also at‐risk of Strep A infections. Overseas studies have noted that the prevalence of Strep C/G throat carriage appears higher in areas where streptococcal disease is hyperendemic. Similar to our findings, Australian research indicates that Strep C/G is rarely isolated from pyoderma or impetigo lesions [6].

The burden of ARF in Aotearoa New Zealand remains a significant public health problem. It is concerning that the national incidence of ARF in 2025 has increased to levels not seen since 2013 [4]. A continued public health emphasis on treating and preventing Strep A infections in high‐risk groups is warranted.

## Funding

This work was supported by Lottery Health Research.

## Ethics Statement

Ethics approval for this study was obtained from the University of Otago, Wellington, New Zealand (Human Ethics Committee reference HD14/12) and the Northern B Health and Disability Ethics Committee, New Zealand (reference 14/NTB/16).

## Conflicts of Interest

The authors declare no conflicts of interest.

## Data Availability

Data may be obtained from a third party and are not publicly available.
